# Performance Analysis for Joint Target Parameter Estimation in UMTS-Based Passive Multistatic Radar with Antenna Arrays Using Modified Cramér-Rao Lower Bounds

**DOI:** 10.3390/s17102379

**Published:** 2017-10-18

**Authors:** Chenguang Shi, Fei Wang, Sana Salous, Jianjiang Zhou

**Affiliations:** 1 Key Laboratory of Radar Imaging and Microwave Photonics, Ministry of Education, Nanjing University of Aeronautics and Astronautics, Nanjing 210016, China; scg_space@163.com (C.S.); zjjee@nuaa.edu.cn (J.Z.); 2 School of Engineering and Computing Sciences, Durham University, Durham DH1 3DE, UK; sana.salous@durham.ac.uk

**Keywords:** modified Cramér-Rao lower bound (MCRLB), modified fisher information matrix (MFIM), maximum likelihood estimation, universal mobile telecommunications system (UMTS) signals, multistatic radar, antenna arrays

## Abstract

In this study, the modified Cramér-Rao lower bounds (MCRLBs) on the joint estimation of target position and velocity is investigated for a universal mobile telecommunication system (UMTS)-based passive multistatic radar system with antenna arrays. First, we analyze the log-likelihood redfunction of the received signal for a complex Gaussian extended target. Then, due to the non-deterministic transmitted data symbols, the analytically closed-form expressions of the MCRLBs on the Cartesian coordinates of target position and velocity are derived for a multistatic radar system with $N_{t}$ UMTS-based transmit station of $L_{t}$ antenna elements and $N_{r}$ receive stations of $L_{r}$ antenna elements. With the aid of numerical simulations, it is shown that increasing the number of receiving elements in each receive station can reduce the estimation errors. In addition, it is demonstrated that the MCRLB is not only a function of signal-to-noise ratio (SNR), the number of receiving antenna elements and the properties of the transmitted UMTS signals, but also a function of the relative geometric configuration between the target and the multistatic radar system.The analytical expressions for MCRLB will open up a new dimension for passive multistatic radar system by aiding the optimal placement of receive stations to improve the target parameter estimation performance.

## 1. Introduction

### 1.1. Background and Motivation

Multiple-input multiple-output (MIMO) radar systems have received contentiously growing attention from the research community in the last decade [1,2], which exploit multiple transmitted waveforms and jointly process the echoes scattered off the target at the multiple receivers. In general, MIMO radar systems can be classified as MIMO radar with widely separated antennas [3] and MIMO radar with colocated antennas [4]. With widely separated transmit and receive antennas, the distributed MIMO radar, sometimes called multistatic radar system or distributed radar network [5,6], can combine the benefits from viewing the targets from different directions. To make the best use of the target information from different directions to achieve high estimation performance, perfect time and space synchronization is required so that all the transmit and receive antennas exploit a common time and space reference in distributed MIMO radar architecture. On the other hand, all the transmit and receive antennas in the colocated MIMO radar are closely spaced, thus providing only a single “view” of the target. Hence, MIMO radar systems with widely distributed antennas can provide improved target parameter estimation capabilities by employing increased geometric diversity [7].

Technically speaking, parameter estimation performance can be assessed by evaluation of bounds on the estimation errors [8]. Cramér-Rao lower bound (CRLB) is one of the most widely used bounds, which can predict the variance of the estimation error when SNR is large or the number of taken data samples is large [9,10]. The CRLBs for distributed MIMO radar systems have been computed for target velocity estimation [11], target location estimation [9], noncoherent and coherent joint target position and velocity estimation [10,12], and multiple-target joint parameter estimation [13]. Recently, He et al. in [14] derive the generalized CRLB for joint estimation of target position and velocity for distributed MIMO radar networks under more general conditions, such as the non-orthogonal signals, spatially dependent target reflection coefficients, and spatially dependent noise. This result is of high importance due to the fact that it describes the best achievable performance for some practical cases. In [15], the CRLBs of the joint time delay and Doppler shift estimation are derived for an extended target, and the effects of transmitted waveform parameters on the CRLBs are analyzed. The authors in [16] investigate the target parameter estimation performance of linear frequency modulation (LFM)-based radar networks in a Rice fading environment. It is shown that the dominant scatterer (DS) component can be exploited to decrease the estimation errors, which is because the reception of DS component increases the received signal-to-noise ratio (SNR) at the radar receiver. Reference [17] derives the CRLB for joint location and velocity estimation of moving target in distributed phased array radars on moving platforms. Numerical examples show that increasing the signal bandwidth is beneficial to improve the location estimation accuracy, while the extended observation time can enhance the velocity estimation accuracy. Furthermore, the target parameter estimation performance is explored in [8] for a radar employing a set of widely separated transmitting and receiving antenna arrays, which considers multiple extended targets under stochastic and deterministic signal model assumptions. It is also worth mentioning that ad-hoc asymptotic performance analysis of algorithms for multistatic localization have appeared in the studies [18,19,20], where the theoretical performance of multiple signal classification (MUSIC) in computational time-reversal (TR) applications is analyzed. The closed-form expression of mean square error (MSE) matrix of TR-MUSIC is calculated for the single-frequency case in both multistatic co-located and non co-located cases, and numerical examples are provided to demonstrate the theoretical results.

### 1.2. Brief Survey of Similar Work

In the last couple of years, extensive research has been conducted in passive radar systems, which utilizes the signals of opportunity as illuminators for target detection [21], parameter estimation [22], target tracking [23], etc. Since passive radars do not require expensive transmission equipments, these systems have the advantages of stealth target detection, low probability of intercept (LPI) [24,25,26,27,28], low implementation cost, anti-jamming [29], and robustness. Further, a passive radar system will offer geometric and signal diversities when it is deployed in a multistatic architecture, which is able to enhance its detection and estimation performance. In [30,31,32,33,34,35,36], the CRLB has been studied and applied to passive radar systems. In [30], the CRLB analysis for the joint target position and velocity estimation is presented for a frequency modulation (FM) based passive radar networks, and it is suggests that more antennas mean better estimation performance. Based on the 3G wireless communications standard, the downlink signal of universal mobile telecommunications systems (UMTS) has been a potential illuminator of opportunity for a passive radar system because of its favourable ambiguity function properties. Moreover, since the transmitted data symbols in UMTS-based waveform are non-deterministic, calculating the classical CRLB is not feasible in this study. In this case, modified CRLB (MCRLB) can provide a looser bound than the classical CRLB in realistic scenarios when computing the classical CRLB is not feasible, which can be employed as a good alternative method by averaging the conventional Fisher information matrix (FIM) conditioned on a given sequence of the transmitted symbols with respect to the stochastic data symbols. Therefore, the MCRLB for UMTS-based passive radar networks is derived in [31], where both noncoherent and coherent modes are considered. However, the studies in [30,31] only investigate the target parameter estimation performance for a passive radar network with widely distributed omnidirectional antennas and do not consider multichannel radar receivers placed on moving platforms. In [32], the results in [31] are extended and the joint target parameter estimation performance of a UMTS-based passive multistatic radar is analyzed in a line-of-sight (LoS) environment. In addition, the MCRLB evaluation of an orthogonal frequency-division multiplexing (OFDM)-based passive radar network can be found in [33], where the OFDM-based L band digital aeronautical communication system type 1 (LDACS1) communication signals are implemented as signals of opportunity for the passive radar networks. The authors in [34] address the target parameter estimation performance of an OFDM-based passive multistatic radar system in a Rice fading environment, which is composed of multiple OFDM-based LDACS1 transmitters of opportunity and multiple radar receivers placed on moving platforms. Reference [35] presents two illumination of opportunity subset selection schemes for FM-based passive radar network configurations, which are formulated as knapsack problems (KPs) and tackled with greedy selection methods. Xie et al. in [36] investigate the problem of joint optimization of receiver placement and transmitter selection for a passive radar network system.

This study addresses the joint target parameter estimation performance of a UMTS-based passive multistatic radar system with antenna arrays, where the multichannel receive stations are placed on moving platforms. The passive multistatic radar system can combine the advantages from geometric diversity, with the benefits of employing standard coherent array processing [8]. It is important to point out that almost all referenced works consider radars with widely distributed omnidirectional antennas and do not consider widely distributed transmitters/receivers with antenna arrays. For the passive multistatic radar system with antenna arrays placed on moving platforms, the things are much more complicated. To the best of our knowledge, there has no study on the problem of joint target parameter estimation in UMTS-based passive multistatic radar system with antenna arrays until now.

### 1.3. Main Contributions

This paper investigates the joint target position and velocity estimation performance for a UMTS-based passive multistatic radar system with antenna arrays, where the multichannel receive stations are placed on moving platforms. It is assumed that each receive station is able to estimate and separate the scattered signals off the target due to different UMTS-based illuminators of opportunity with perfect accuracy. Owing to the stochastic parameters describing the UMTS waveform, the MCRLB is utilized as an alternative to the classical CRLB.

The major contributions of this work are summarized as follows:

(1) We formulate the signal model and derive the log-likelihood function for a multistatic radar system with $N_{t}$ UMTS-based transmit stations of $L_{t}$ antenna elements and $N_{r}$ receive stations of $L_{r}$ antenna elements. As aforementioned, it is noteworthy that references [30,31,32,33,34] only evaluate the target parameter estimation performance for a passive multistatic radar with widely distributed omnidirectional antennas and do not consider receive stations placed on moving platforms. While Khomchuk et al. in [8] concentrate on stationary platforms, and the passive multistatic radar system is ignored in [8,17]. As an extension, this paper is a much more generalized case and quite different from the obtained results in [8,17,30,31,32,33,34].
(2) The analytically closed-form expressions of MCRLB on the joint estimation of target position and velocity are derived in the presence of the nuisance parameters. These expressions for MCRLB can be used as a unified performance metric in that they enable the optimal placement of receive stations to improve the target parameter estimation accuracy.
(3) The obtained numerical simulation results suggest that increasing the number of receiving elements in each receive station can reduce the estimation errors. Previous results in [31,32] only demonstrate that the CRLB is a function of the properties of the transmitted signals and the geometric configuration between the target and the distributed MIMO radar systems. Herein, the effects of SNR and the number of receiving antenna elements on the joint target position and velocity estimation performance are also discussed. Specifically, it is shown that the joint MCRLB is a function of SNR, the number of receiving antenna elements, the UMTS signal parameters, as well as the relative geometric architecture between the target and the passive multistatic radar systems.

Note that the objective of a typical UMTS-based communication system is to transfer information from a source to a sink and recover that information reliably. While the passive multistatic radar system uses the signals transmitted from the UMTS-based illuminators of opportunity to detect and estimate targets, which makes it a potential technology for LPI and other as aforementioned advantages. Thus, modifying the transmitted UMTS waveform parameters to maintain LPI performance may be out of the scope of the UMTS-based communication systems and will not be discussed in this paper.

### 1.4. Outline of the Paper

The remainder of this paper is organized as follows. The signal model for UMTS-based passive multistatic radar with antenna arrays is discussed in Section 1, in which each receive station employs coherent processing. The log-likelihood function is derived and the maximum likelihood estimation (MLE) is analyzed in Section 2. In Section 3, the closed-form expressions of MCRLB for target parameter estimation are computed. Numerical simulations and performance analysis are presented in Section 4. Finally, our concluding remarks are summarized in Section 5.

**Notation:** The superscript *T* represents the transpose operator; E{·} and ${\left(\cdot \right)}^{*}$ represent the expectation and conjugation operators, respectively. |·| denotes the absolute value, ℜ{·} is the real part, and ℑ{·} is the imaginary part. ⊙ represents the Hardmard product, ⊗ represents the Kronecker product. $U_{i}\left(f\right)$ denotes the Fourier transform of $u_{i}\left(t\right)$.

## 2. Signal Model

Let us consider a passive multistatic radar system with $N_{t}$ widely spaced transmit stations and $N_{r}$ widely spaced receive stations. Each transmit and receive station consists of $L_{t}$ and $L_{r}$ antenna elements respectively. Here, the receive stations can receive echoes from the target due to the UMTS signals scattered off the target as well as the UMTS signals from the transmit stations whose locations are supposed to be known. The UMTS signals are received via two directions: a path which is due to scattering off the target and a direct path. It is also assumed that the passive receive stations can employ adaptive beamforming to separate the received signals into two channels, one required for target surveillance and the other for receiving the reference UMTS signals through the direct path. Adaptive beamforming is a widely utilized technique in radar and wireless communication systems, which is able to reject interferences from other angles. In addition, successive interference cancellation (SIC) is used to remove a stronger reference UMTS signal from the observed signal in order to obtain interference-free target return. As such, the UMTS signals from different transmit stations can be estimated and separated at the receive stations (for example different frequency spectra) [31]. The center of the *i*-th transmit station is located at $\vec{p_{i}^{t}}=\left[x_{i}^{t},y_{i}^{t}\right]$ , $i=1,\cdots ,N_{t}$, while the center of the *j*-th receive station is located at $\vec{p_{j}^{r}}=\left[x_{j}^{r},y_{j}^{r}\right]$, $j=1,\cdots ,N_{r}$. The target position and velocity are supposed to be deterministic unknown and denoted by $\vec{p}=\left[x,y\right]$ and $\vec{v}=\left[v_{x},v_{y}\right]$. The unknown target state vector that collects the parameters to be estimated can be defined as follows:

(1) $$\begin{array}{c} \Phi ={\left[x,y,v_{x},v_{y}\right]}^{T}, \end{array}$$

Let $\tau_{ij}^{\Phi }$ represent the bistatic time delay corresponding to the path between the *i*-th transmit station, moving target, and the *j*-th receive station, which is a function of the unknown target position $\vec{p}=\left[x,y\right]$:

(2) $$\begin{array}{cc} \tau_{ij}^{\Phi } & =\frac{\sqrt{{\left(x-x_{i}^{t}\right)}^{2}+{\left(y-y_{i}^{t}\right)}^{2}}+\sqrt{{\left(x-x_{j}^{r}\right)}^{2}+{\left(y-y_{j}^{r}\right)}^{2}}}{c}\\ & =\frac{∥\vec{p}-\vec{p_{i}^{t}}∥+∥\vec{p}-\vec{p_{j}^{r}}∥}{c_{v}}, \end{array}$$

where $c_{v}$ is the speed of light, $∥\vec{p}-\vec{p_{i}^{t}}∥$ denotes the distance from the *i*-th transmit station to the target and $∥\vec{p}-\vec{p_{j}^{r}}∥$ denotes the distance from the target to the *j*-th receive station, respectively. In this paper, the receive stations are placed on moving platforms. The *j*th receive station is moving with velocity $\vec{v_{j}^{r}}=\left[v_{x,j}^{r},v_{y,j}^{r}\right]$. With the aforementioned positions/velocities of the target and receive stations, the Doppler shift of the moving target corresponding to the ij-th path is the time rate of change of the total ij-th path length:

(3) $$\begin{array}{cc} f_{D_{ij}}^{\Phi } & =\frac{1}{\lambda }\left[\frac{\partial ∥\vec{p}-\vec{p_{i}^{t}}∥}{\partial t}+\frac{\partial ∥\vec{p}-\vec{p_{j}^{r}}∥}{\partial t}\right]\\ & =\frac{1}{\lambda }\left[v_{x}\left(\frac{x-x_{i}^{t}}{∥\vec{p}-\vec{p_{i}^{t}}∥}+\frac{x-x_{j}^{r}}{∥\vec{p}-\vec{p_{j}^{r}}∥}\right)\right]+\frac{1}{\lambda }\left[v_{y}\left(\frac{y-y_{i}^{t}}{∥\vec{p}-\vec{p_{i}^{t}}∥}+\frac{y-y_{j}^{r}}{∥\vec{p}-\vec{p_{j}^{r}}∥}\right)\right]\\ & +\frac{1}{\lambda }\left[v_{x,j}^{r}\frac{x-x_{j}^{r}}{∥\vec{p}-\vec{p_{j}^{r}}∥}+v_{y,j}^{r}\frac{y-y_{j}^{r}}{∥\vec{p}-\vec{p_{j}^{r}}∥}\right], \end{array}$$

where λ denotes the carrier wavelength, $\frac{\partial ∥\vec{p}-\vec{p_{i}^{t}}∥}{\partial t}$ and $\frac{\partial ∥\vec{p}-\vec{p_{j}^{r}}∥}{\partial t}$ are the relative velocities for the *i*-th transmit station and the *j*-th receive station, respectively. One can notice from Equation (2) that the Doppler shift $f_{D_{ij}}^{\Phi }$ is a function of the unknown target position $\vec{p}=\left[x,y\right]$ and velocity $\vec{v}=\left[v_{x},v_{y}\right]$.

The lowpass equivalent waveform transmitted from the *i*-th UMTS-based transmit station is $\sqrt{\frac{E}{N_{t}L_{t}}}u_{i}\left(t\right)$, where *E* denotes the total transmitted energy. The baseband signal corresponding to the *i*-th transmit station $u_{i}\left(t\right)$ is normalized $\int_{-\infty }^{+\infty }{\left|u_{i}\left(t\right)\right|}^{2}dt=1$, which is defined as follows:

(4) $$\begin{array}{c} u_{i}\left(t\right)=\frac{1}{\sqrt{N}}\sum_{n=0}^{N-1}c_{in}g_{i}\left(t-nT\right), \end{array}$$

where *N* is the total number of symbols, $c_{in}$ is the *i*-th transmitted quadrature phase shift keying (QPSK) symbol, and *T* is the symbol time. The symbols are modulated with delayed root-raised-cosine (RRC) pulse $g_{i}\left(t\right)=h_{i}\left(t-\frac{D}{2}\right)$, where the definitions of the delay *D* and $h_{i}\left(t\right)$ can be found in [32].

**Assumption** **1.** *Suppose that the transmitted signals are approximately orthogonal*

(5) $$\begin{array}{c} \int_{-\infty }^{+\infty }u_{i}\left(t\right)u_{i^{\prime }}^{*}\left(t\right)dt\approx \left\{\begin{array}{cc} 1, & \;\;\mathrm{if}\;i=i^{\prime }\\ 0, & \;\;\mathrm{if}\;i\neq i^{\prime } \end{array}\right. \end{array}$$

and maintain approximate orthogonal for time delays $\tau_{ij}^{\Phi }$, $\tau_{i^{\prime }j^{\prime }}^{\Phi }$ and Doppler shifts $f_{D_{ij}}^{\Phi }$, $f_{D_{i^{\prime }j^{\prime }}}^{\Phi }$ of interest

(6) $$\begin{array}{c} \int_{-\infty }^{+\infty }u_{i}\left(t-\tau_{ij}^{\Phi }\right)u_{i^{\prime }}^{*}\left(t-\tau_{i^{\prime }j^{\prime }}^{\Phi }\right)e^{j2\pi (f_{D_{ij}}^{\Phi }-f_{D_{i^{\prime }j^{\prime }}}^{\Phi })t}dt\approx \left\{\begin{array}{cc} 1, & \;\;\mathrm{if}\;i=i^{\prime },\;j=j^{\prime }\\ 0, & \;\;\mathrm{if}\;i\neq i^{\prime },\;j\neq j^{\prime } \end{array}\right. \end{array}$$

such that the signals from different transmit stations can be separated at each receive station [10].

**Assumption** **2.** The transmit and receive stations are sufficiently separated so that each transmit-to-receive path provides an independent aspect angle for the target, such that each of them has its own reflection coefficient.

Under these assumptions, the signal received at the *k*-th antenna element of the *j*-th receive station is given by:

(7) $$\begin{array}{c} r_{jk}\left(t\right)=\sqrt{\frac{E}{N_{t}L_{t}}}\sum_{i=1}^{N_{t}}\xi_{ij}Gu_{i}\left(t-\tau_{ij}^{\Phi }\right)e^{j2\pi f_{D_{ij}}^{\Phi }t}e^{j2\pi \left(k-1\right)sin\phi_{j}^{r}d_{r}/\lambda }+w_{jk}\left(t\right), \end{array}$$

where $\xi_{ij}=\xi_{ijR}+j\xi_{ijI}$ denotes the complex reflection coefficient corresponding to the ij-th path, which is unknown parameter. Define a target reflection vector that collects reflection coefficients for all paths as:

(8) $$\begin{array}{c} \xi ={\left[\xi_{11},\xi_{21},\cdots ,\xi_{N_{t}1},\xi_{21},\cdots ,\xi_{N_{t}N_{r}}\right]}^{T}, \end{array}$$

and it is assumed that the reflection coefficient corresponding to the ij-th path $\xi_{ij}$ is a complex Gaussian distributed variable with zero mean and covariance $\sigma_{\xi }^{2}$, i.e., $\xi_{ij}∼\mathrm{CN}\left(0,\sigma_{\xi }^{2}\right)$. *G* denotes the antenna gain of the transmit station, $\phi_{j}^{r}=\arctan\left[\left(y_{j}^{r}-y\right)/\left(x_{j}^{r}-x\right)\right]$, $d_{r}$ denotes the element spacing of receive station. The term $w_{jk}\left(t\right)$ represents noise at the *k*-th antenna element of the *j*-th receive station, which is supposed to be independent and identically-distributed zero-mean complex Gaussian with variance $\sigma_{w}^{2}$, i.e., $w_{jk}∼\mathrm{CN}\left(0,\sigma_{w}^{2}\right)$. The $L_{r}$ observations of the *j*-th receive station can be expressed as [17]:

(9) $$\begin{array}{c} r_{j}\left(t\right)={\left[r_{j1}\left(t\right),\cdots ,r_{jk}\left(t\right),\cdots ,r_{jL_{r}}\left(t\right)\right]}^{T}. \end{array}$$

The observations from all $M_{r}$ receive stations can be written as follows:

(10) $$\begin{array}{c} r\left(t\right)={\left[r_{1}^{T}\left(t\right),\cdots ,r_{j}^{T}\left(t\right),\cdots ,r_{M_{r}}^{T}\left(t\right)\right]}^{T}, \end{array}$$

which collects the observed signals from the entire set of the receiving antenna elements.

**Remark** **1.** *To provide an accurate estimation of the moving target, the installation of radar nodes in a multistatic radar system require that the transmit and receive stations demand time synchronization to perform correct target parameter estimation. Herein, it is assumed that the time synchronization of the multistatic radar is achieved by global positioning system (GPS), which can provide precise time stamps (up to nanoseconds) at both transmit and receive stations.*

## 3. Maximum Likelihood Estimation

Under Assumptions 1 and 2, and suppose that the noise and the target reflection coefficients are mutually independent, the joint probability density function (PDF) of the received signals r(t) for a given transmitted symbol vector c can be expressed as:

(11) $$p\left(r\left(t\right)|\Phi ,\xi ,c\right)\propto \exp\left\{-\frac{1}{\sigma_{w}^{2}}\sum_{j=1}^{N_{r}}\sum_{k=1}^{L_{r}}\int_{-\infty }^{+\infty }{\left|r_{jk}\left(t\right)-\sqrt{\frac{E}{N_{t}L_{t}}}\sum_{i=1}^{N_{t}}\xi_{ij}Gu_{i}\left(t-\tau_{ij}^{\Phi }\right)e^{j2\pi f_{D_{ij}}^{\Phi }t}e^{j2\pi \left(k-1\right)sin\phi_{j}^{r}d_{r}/\lambda }\right|}^{2}dt\right\}.$$

where c represents a vector that collects any random parameters needed to describe the UMTS-based waveform. Furthermore, taking logarithm of the pdf in Equation (11), we can obtain the log-likelihood function as follows:

(12) $$\begin{array}{cc} L(r(t)|\Phi ,\xi ,c) & =\ln p(r(t)|\Phi ,\xi ,c)\\ & =-\frac{1}{\sigma_{w}^{2}}\sum_{j=1}^{N_{r}}\sum_{k=1}^{L_{r}}\int_{-\infty }^{+\infty }{\left|r_{jk}\left(t\right)-\sqrt{\frac{E}{N_{t}L_{t}}}\sum_{i=1}^{N_{t}}\xi_{ij}Gu_{i}\left(t-\tau_{ij}^{\Phi }\right)e^{j2\pi f_{D_{ij}}^{\Phi }t}e^{j2\pi \left(k-1\right)sin\phi_{j}^{r}d_{r}/\lambda }\right|}^{2}dt+C, \end{array}$$

where C is not dependent on the target state vector Φ and reflection vector ξ.

Since the target reflection vector ξ is unknown, the log-likelihood function lnp(r(t)|Φ,c) can be obtained by MLE. To be specific, the complex reflection coefficient of the ij-th transmit-to-receive path maximizes L(r(t)|Φ,ξ,c) in Equation (12), i.e.,

(13) $$\left\{\begin{array}{c} \frac{\partial }{\partial \xi_{ijR}}L\left(r\left(t\right)|\Phi ,\xi ,c\right){|}_{\xi_{ijR}=\hat{\xi_{ijR}}}=0,\\ \frac{\partial }{\partial \xi_{ijI}}L\left(r\left(t\right)|\Phi ,\xi ,c\right){|}_{\xi_{ijI}=\hat{\xi_{ijI}}}=0. \end{array}\right.$$

Then, the maximum likelihood (ML) estimates of $\hat{\xi_{ijR}}$ and $\hat{\xi_{ijI}}$ can be obtained as:

(14) $$\left\{\begin{array}{c} \hat{\xi_{ijR}}=\frac{ℜ\left\{\sum_{k=1}^{L_{r}}\int_{-\infty }^{+\infty }r_{jk}\left(t\right)u_{i}^{*}\left(t-\tau_{ij}^{\Phi }\right)e^{-j2\pi f_{D_{ij}}^{\Phi }t}e^{-j2\pi \left(k-1\right)sin\phi_{j}^{r}d_{r}/\lambda }dt\right\}}{\sqrt{\frac{E}{N_{t}L_{t}}}L_{r}G},\\ \hat{\xi_{ijI}}=\frac{ℑ\left\{\sum_{k=1}^{L_{r}}\int_{-\infty }^{+\infty }r_{jk}\left(t\right)u_{i}^{*}\left(t-\tau_{ij}^{\Phi }\right)e^{-j2\pi f_{D_{ij}}^{\Phi }t}e^{-j2\pi \left(k-1\right)sin\phi_{j}^{r}d_{r}/\lambda }dt\right\}}{\sqrt{\frac{E}{N_{t}L_{t}}}L_{r}G}, \end{array}\right.$$

Thus, the estimated target reflection coefficient corresponding to the ij-th path can be expressed as:

(15) $$\begin{array}{cc} \hat{\xi_{ij}} & =\hat{\xi_{ijR}}+j\hat{\xi_{ijI}}\\ & =\frac{\sum_{k=1}^{L_{r}}\int_{-\infty }^{+\infty }r_{jk}\left(t\right)u_{i}^{*}\left(t-\tau_{ij}^{\Phi }\right)e^{-j2\pi f_{D_{ij}}^{\Phi }t}e^{-j2\pi \left(k-1\right)sin\phi_{j}^{r}d_{r}/\lambda }dt}{\sqrt{\frac{E}{N_{t}L_{t}}}L_{r}G} \end{array}$$

Substituting $\hat{\xi_{ij}}$ back into Equation (12) instead of the corresponding variables, the concentrated log-likelihood function lnp(r(t)|Φ,c) can be written as:

(16) $$\begin{array}{cc} L(r(t)|\Phi ,c) & =\ln p(r(t)|\Phi ,c)\\ & =-\frac{1}{\sigma_{w}^{2}}\sum_{j=1}^{N_{r}}\sum_{k=1}^{L_{r}}\int_{-\infty }^{+\infty }{\left|r_{jk}\left(t\right)\right|}^{2}dt\\ & +\frac{1}{L_{r}\sigma_{w}^{2}}\sum_{i=1}^{N_{t}}\sum_{j=1}^{N_{r}}{\left|\sum_{k=1}^{L_{r}}\int_{-\infty }^{+\infty }r_{jk}\left(t\right)u_{i}^{*}\left(t-\tau_{ij}^{\Phi }\right)e^{-j2\pi f_{D_{ij}}^{\Phi }t}e^{-j2\pi \left(k-1\right)sin\phi_{j}^{r}d_{r}/\lambda }dt\right|}^{2}, \end{array}$$

From a practical stand point, it is assumed that the vector c is perfectly known or estimated after a previous estimation step [37]. Subsequently, neglecting the constant term of the second line in Equation (16), the estimate of the unknown target state vector Φ can be found as the minimizer of the function

(17) $$\begin{array}{cc} \hat{\Phi_{\mathrm{ML}}} & =\arg\max_{\Phi }L\left(r\left(t\right)|\Phi ,c\right)\\ & =\arg\max_{\Phi }\frac{1}{L_{r}\sigma_{w}^{2}}\sum_{i=1}^{N_{t}}\sum_{j=1}^{N_{r}}{\left|\sum_{k=1}^{L_{r}}\int_{-\infty }^{+\infty }r_{jk}\left(t\right)u_{i}^{*}\left(t-\tau_{ij}^{\Phi }\right)e^{-j2\pi f_{D_{ij}}^{\Phi }t}e^{-j2\pi \left(k-1\right)sin\phi_{j}^{r}d_{r}/\lambda }dt\right|}^{2}, \end{array}$$

where $\hat{\Phi_{\mathrm{ML}}}$ represents the MLE of the unknown parameter vector Φ.

**Remark** **2.** *It is worth pointing out that a four-dimensional search over the space consisting of the possible values of $\left(x,y,v_{x},v_{y}\right)$ is required for obtaining the ML estimate of* **Φ** *numerically. Additionally, as the numbers of $N_{t}$, $N_{r}$, and $L_{r}$ go up, the system complexity and computational load will be increased significantly. There are some suboptimal methods which can achieve low complexity [38,39]. In [38], a reduced complexity optimal algorithm is presented, which employs two dimensional fast Fourier transform to jointly estimate the target position and Doppler shift. It is also shown that the MSE of the estimators achieves the CRLB. In [39], the first-order Keystone transform and the Lv’s transform are utilized to estimate the multiple targets’ motion parameters, which is fast and can obtain the accurate parameter estimation without knowing the number of targets and their motion information. Future work will develop some other suboptimal approaches to reduce complexity.*

## 4. Derivation of Modified Cramér-Rao Lower Bound

In this section, we will derive the MCRLB for jointly estimating the target position (x,y) and velocity $\left(v_{x},v_{y}\right)$ for a multistatic radar system with $N_{t}$ UMTS-based transmit station of $L_{t}$ antenna elements and $N_{r}$ receive stations of $L_{r}$ antenna elements. Generally speaking, the CRLB is a good prediction of the variance of the estimation error due to the fact that the CRLB is close to the MSE of the MLE when SNR is large or the number of taken data samples is large. However, since the transmitted data symbols $c_{in}$ are non-deterministic, calculating the classical CRLB is not feasible in our work, which represent nuisance parameters in the estimation process. In this case, MCRLB can provide a good benchmark when computing the classical CRLB is not feasible. In the classical CRLB, the joint probability density function of the received signal and the parameter vector is used, while in MCRLB the expectation is taken on the conditioned probability density function of the received signal conditioned on the transmitted symbols [34]. The expressions of MCRLB can be employed as a good alternative method by averaging the conventional FIM conditioned on a given sequence of the transmitted symbols with respect to the stochastic data symbols, which show a looser bound than the classical CRLB in realistic scenarios. The first step in deriving MCRLB is to compute the modified FIM (MFIM), which is a 4×4 matrix obtained from the second-order derivatives of the concentrated log-likelihood function:

(18) $$\begin{array}{cc} J(\Phi |c) & =E_{r(t)|\Phi ,c}\left\{{▽}_{\Phi }L\left(r\left(t\right)|\Phi ,c\right){\left[{▽}_{\Phi }L\left(r\left(t\right)|\Phi ,c\right)\right]}^{T}\right\}\\ & =-E_{r(t)|\Phi ,c}\left\{{▽}_{\Phi }{\left[{▽}_{\Phi }L\left(r\left(t\right)|\Phi ,c\right)\right]}^{T}\right\}, \end{array}$$

We can observe from Equation (16) that L(r(t)|Φ,c) is a function of $\tau_{ij}^{\Phi }$ and $f_{D_{ij}}^{\Phi }$, thus we define an intermediate parameter vector:

(19) $$\begin{array}{cc} \Psi & ={\left[\tau_{11}^{\Phi },\tau_{12}^{\Phi },\cdots ,\tau_{N_{t}N_{r}}^{\Phi },f_{D_{11}}^{\Phi },f_{D_{12}}^{\Phi },\cdots ,f_{D_{N_{t}N_{r}}}^{\Phi }\right]}^{T}, \end{array}$$

which collects the unknown time delays and Doppler shifts. According to the chain rule, the MFIM can be computed by:

(20) $$\begin{array}{cc} J(\Phi |c) & =\left({▽}_{\Phi }\Psi^{T}\right)J\left(\Psi |c\right){\left({▽}_{\Phi }\Psi^{T}\right)}^{T}, \end{array}$$

where $J\left(\Psi |c\right)=-E_{r(t)|\Psi ,c}\left\{{▽}_{\Psi }L\left(r\left(t\right)|\Psi ,c\right){\left[{▽}_{\Psi }L\left(r\left(t\right)|\Psi ,c\right)\right]}^{T}\right\}$.

First, we compute ${▽}_{\Phi }\Psi^{T}$. Recalling Equation (1) and (19) , ${▽}_{\Phi }\Psi^{T}$ can be obtained as:

(21) $${▽}_{\Phi }\Psi^{T}=\left[\begin{array}{cc} \Omega^{1} & \Omega^{2}\\ 0 & \Omega^{4} \end{array}\right],$$

where

(22) $$\Omega^{1}=\left[\begin{array}{cccc} \frac{\partial \tau_{11}^{\Phi }}{\partial x} & \frac{\partial \tau_{12}^{\Phi }}{\partial x} & \cdots & \frac{\partial \tau_{N_{t}N_{r}}^{\Phi }}{\partial x}\\ \frac{\partial \tau_{11}^{\Phi }}{\partial y} & \frac{\partial \tau_{12}^{\Phi }}{\partial y} & \cdots & \frac{\partial \tau_{N_{t}N_{r}}^{\Phi }}{\partial y} \end{array}\right],$$

(23) $$\Omega^{2}=\left[\begin{array}{cccc} \frac{\partial f_{D_{11}}^{\Phi }}{\partial x} & \frac{\partial f_{D_{12}}^{\Phi }}{\partial x} & \cdots & \frac{\partial f_{D_{N_{t}N_{r}}}^{\Phi }}{\partial x}\\ \frac{\partial f_{D_{11}}^{\Phi }}{\partial y} & \frac{\partial f_{D_{12}}^{\Phi }}{\partial y} & \cdots & \frac{\partial f_{D_{N_{t}N_{r}}}^{\Phi }}{\partial y} \end{array}\right],$$

(24) $$\Omega^{4}=\left[\begin{array}{cccc} \frac{\partial f_{D_{11}}^{\Phi }}{\partial v_{x}} & \frac{\partial f_{D_{12}}^{\Phi }}{\partial v_{x}} & \cdots & \frac{\partial f_{D_{N_{t}N_{r}}}^{\Phi }}{\partial v_{x}}\\ \frac{\partial f_{D_{11}}^{\Phi }}{\partial v_{y}} & \frac{\partial f_{D_{12}}^{\Phi }}{\partial v_{y}} & \cdots & \frac{\partial f_{D_{N_{t}N_{r}}}^{\Phi }}{\partial v_{y}} \end{array}\right],$$

and 0 is a $2\times N_{t}N_{r}$ zero matrix. The entries of $\Omega^{1}$, $\Omega^{2}$, and $\Omega^{4}$ are provided in Appendix A.

Then, we derive J(Ψ|c), which is a $2N_{t}N_{r}\times 2N_{t}N_{r}$ matrix and can be rewritten as a block matrix:

(25) $$J\left(\Psi |c\right)=\left[\begin{array}{cc} J{\left(\Psi |c\right)}^{UL} & J{\left(\Psi |c\right)}^{UR}\\ J{\left(\Psi |c\right)}^{LL} & J{\left(\Psi |c\right)}^{LR} \end{array}\right],$$

The detailed derivations of $J{\left(\Psi |c\right)}^{UL}$, $J{\left(\Psi |c\right)}^{UR}$, $J{\left(\Psi |c\right)}^{LL}$, and $J{\left(\Psi |c\right)}^{LR}$ are shown in Appendix B.

After lengthy algebraic calculations, the final expression for total MFIM across all the transmit-to-receive paths can be written as follows:

(26) $$\begin{array}{c} J\left(\Phi |c\right)=\sum_{i=1}^{N_{t}}\sum_{j=1}^{N_{r}}\frac{8\pi^{2}L_{r}EG^{2}{\left|\xi_{ij}\right|}^{2}}{N_{r}L_{t}\sigma_{w}^{2}}J_{ij}\left(\Phi |c\right), \end{array}$$

The expressions for the elements of the bistatic MFIM $J_{ij}\left(\Phi |c\right)$ corresponding to the ij-th transmit-to-receive path are presented in Appendix C. The MCRLB for the unknown target state vector Φ is defined as an inverse of the MFIM:

(27) $$\begin{array}{c} \mathrm{CRLB}\left(\Phi |c\right)=J^{-1}\left(\Phi |c\right). \end{array}$$

One can observe from Equation (26) that the MCRLB CRLB(Φ|c) is a summation of $N_{t}N_{r}$ terms, such that each transmit-to-receive path contributes information about the target’s parameters of interest [8]. The joint MCRLBs for the estimates of the unknown target position and velocity components can be determined by the diagonal elements of the inverse of the MFIM evaluated at the true parameter value:

(28) $$\left\{\begin{array}{c} \mathrm{var}\left(\hat{x}\right)\geq {\left[J^{-1}\left(\Phi |c\right)\right]}_{1,1},\\ \mathrm{var}\left(\hat{y}\right)\geq {\left[J^{-1}\left(\Phi |c\right)\right]}_{2,2},\\ \mathrm{var}\left(\hat{v_{x}}\right)\geq {\left[J^{-1}\left(\Phi |c\right)\right]}_{3,3},\\ \mathrm{var}\left(\hat{v_{y}}\right)\geq {\left[J^{-1}\left(\Phi |c\right)\right]}_{4,4}. \end{array}\right.$$

where $\mathrm{var}(\hat{\vartheta })$ denotes the variance of any unbiased estimation $\hat{\vartheta }$ of the unknown parameter θ. Since the closed-form expression of $J^{-1}\left(\Phi |c\right)$ can be easily derived employing the chain rule, the MCRLB can be computed for any nonsingular MFIM. It should be noted that the computational complexity of the MCRLB computation will be increased as we increase the numbers of transmit and receive stations, while the size of J(Φ|c) does not change with $N_{t}$ and $N_{r}$ [10]. In Section 4, examples are dedicated to calculate the joint MCRLB for a UMTS-based passive multistatic radar system with antenna arrays as well as reveal the effects of several factors on the MCRLB.

**Remark** **3.** Without loss of generality, we focus on a single-target case in this paper. However, the results can be extended to the multiple-target scenario, in which the number of unknown parameters in the target state vector is increased by a factor equal to the number of targets, that is, $\Phi ={\left[x^{1},y^{1},v_{x}^{1},v_{y}^{1},\cdots ,x^{Q},y^{Q},v_{x}^{Q},v_{y}^{Q}\right]}^{T}$, where Q denotes the total number of targets. The intermediate target parameter vector in Equation (19) can be rewritten as $\Psi ={\left[\tau_{11,1}^{\Phi },\cdots ,\tau_{N_{t}N_{r},1}^{\Phi },\cdots ,\tau_{11,Q}^{\Phi },\cdots ,\tau_{N_{t}N_{r},Q}^{\Phi },f_{D_{11},1}^{\Phi },\cdots ,f_{D_{N_{t}N_{r}},1}^{\Phi },\cdots ,f_{D_{11},Q}^{\Phi },\cdots ,f_{D_{N_{t}N_{r}},Q}^{\Phi }\right]}^{T}$. Then, ${▽}_{\Phi }\Psi^{T}$ becomes a $4Q\times 2QN_{t}N_{r}$ matrix, and J(Ψ|c) is a $2QN_{t}N_{r}\times 2QN_{t}N_{r}$ matrix. Thus, the final closed-form expressions for MCRLB will be a 4Q×4Q matrix. It is indicated in [13] that the interactions between different targets can be ignored when the distances between the targets are large enough. Therefore, each target can be treated separately as it is treated in the problem of single-target parameter estimation.

## 5. Numerical Simulations and Performance Analysis

In this section, numerical examples are provided to compute the MCRLB for a UMTS-based passive multistatic radar system with antenna arrays, which demonstrate the use of the derived MCRLB to bound the performance of joint target position and velocity estimation.

### 5.1. Numerical Setup

**Figure 1:**
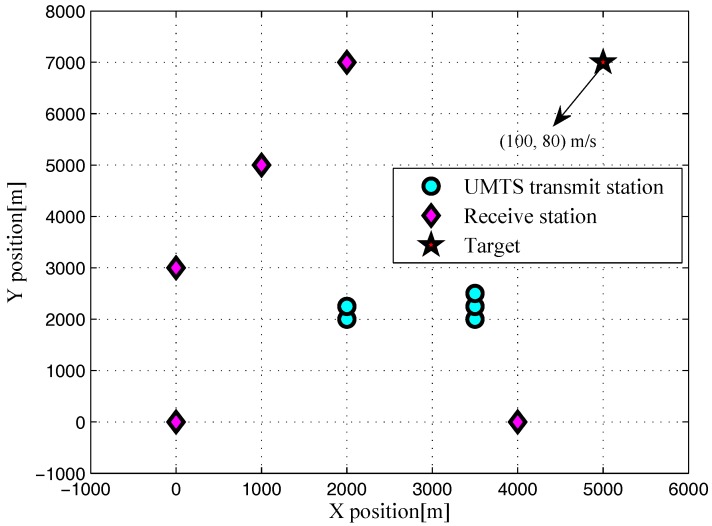
Simulated multistatic 2D scenario with locations of transmit stations, receive stations and target.

For numerical simulations, we assume a multistatic radar system with five UMTS-based transmit station and an equal number of receive stations, i.e., $N_{t}=5$ and $N_{r}=5$. The Cartesian coordinates of the positions of transmit stations (in m) are given in Table 1. The positions and moving parameters of the receive stations are provided in Table 2. Consider a target moving with velocity [100,80]m/s is located at [5000,7000]m. For a brief discussion, we consider a scenario in a 2D geometry as visualized in Figure 1. In order to compare different radar systems based on the same amount of transmitted energy and signal bandwidth, it is assumed that all UMTS-based transmit stations considered in this section have single element transmitting arrays, i.e., $L_{t}=1$. For the simulation parameters, we choose T=0.26μs, N=1024, α=0.22, and the center frequency $f_{c}=2100\;\mathrm{MHz}$ as in [31,32].

### 5.2. Simulation Results

As aforementioned, the reflection coefficient corresponding to the ij-th path $\xi_{ij}$ is modelled as a zero-mean complex Gaussian random variable with variance $\sigma_{\xi }^{2}$, i.e., $\xi_{ij}∼\mathrm{CN}\left(0,\sigma_{\xi }^{2}\right)$. Define the SNR as:

(29) $$\begin{array}{c} \mathrm{SNR}=10\mathrm{lg}\left(\frac{EG^{2}\sigma_{\xi }^{2}}{N_{t}L_{t}\sigma_{w}^{2}}\right). \end{array}$$

**Figure 2:**
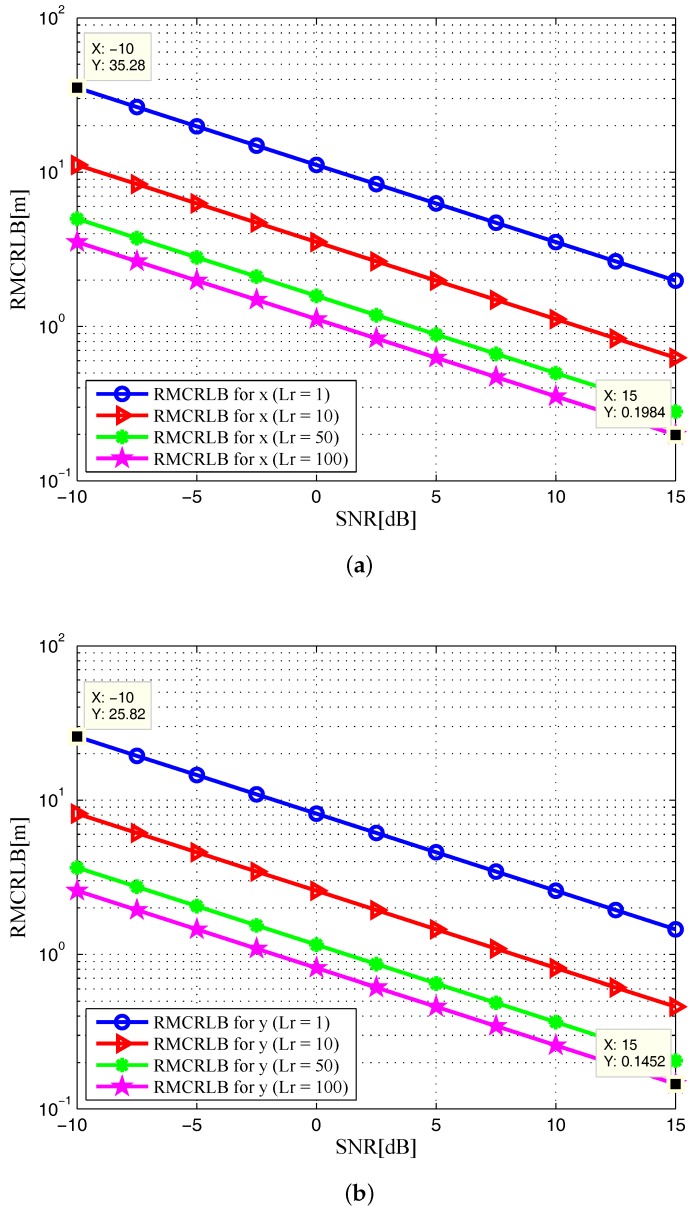
RMCRLB versus SNR in target position dimensions with different $L_{r}$: (**a**) x-position; (**b**) y-position.

**Figure 3:**
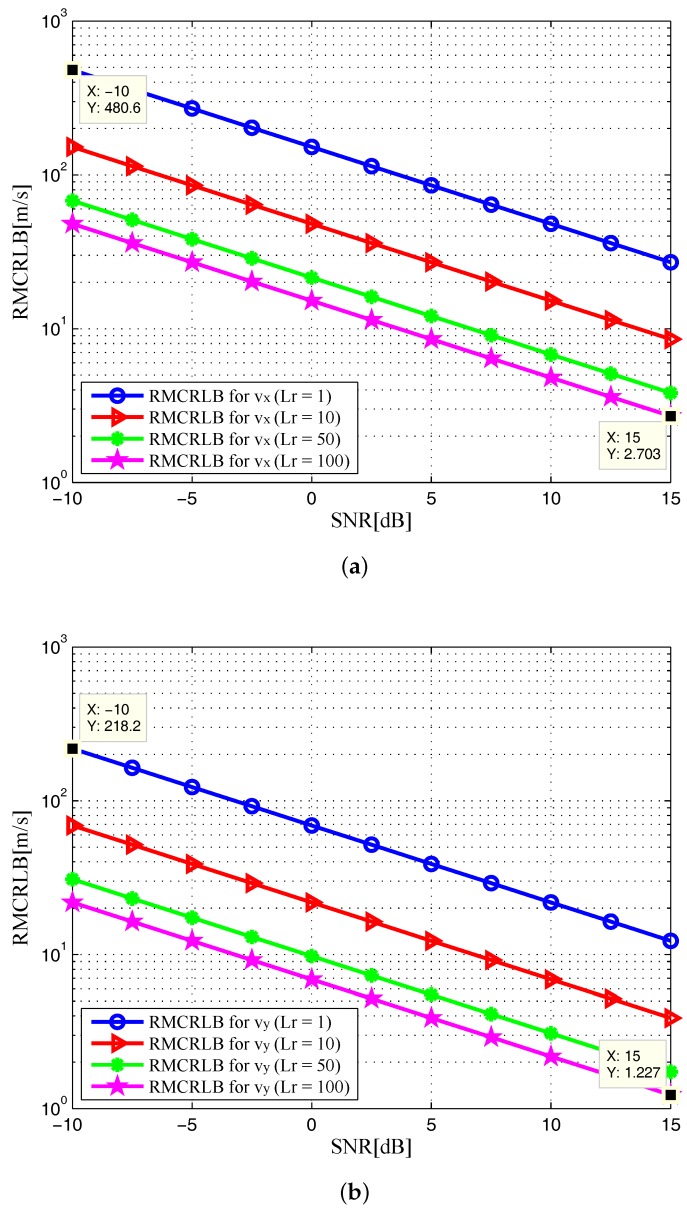
RMCRLB versus SNR in target velocity dimensions with different $L_{r}$: (**a**) x-velocity; (**b**) y-velocity.

In Figure 2, the noncoherent square roots of MCRLB (RMCRLBs) are plotted against SNR for x-position and y-position dimensions. Similarly, we plot the velocity RMCRLBs for varying SNR in Figure 3. One can see that the RMCRLBs for both target position and velocity estimates decrease with an increase of SNR. It is obvious from Figure 2 and Figure 3 that as the value of $L_{r}$ goes up, the estimation errors are decreased significantly for Cartesian components of target position and velocity, which demonstrates that grouping the receiving elements into properly sized arrays can reduce MSE [8].

**Figure 4:**
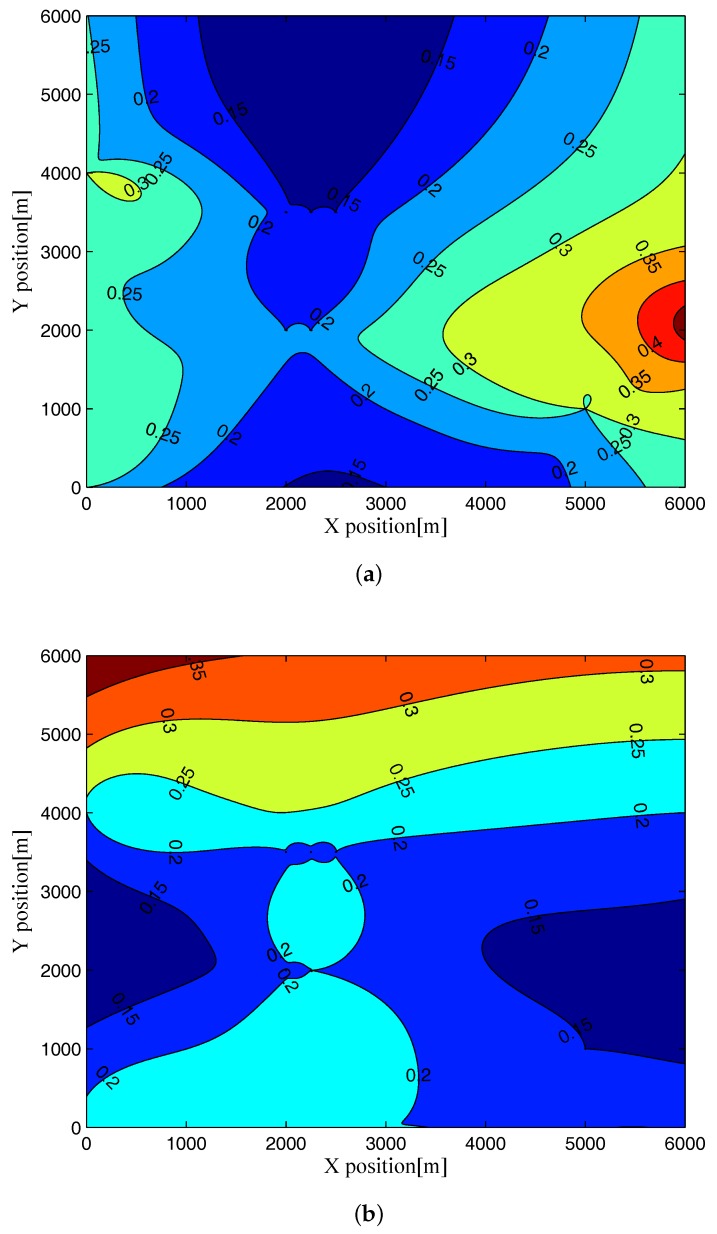
RMCRLB for target position dimensions in different position when SNR=0dB and $L_{r}=1000$: (**a**) x-position; (**b**) y-position.

**Figure 5:**
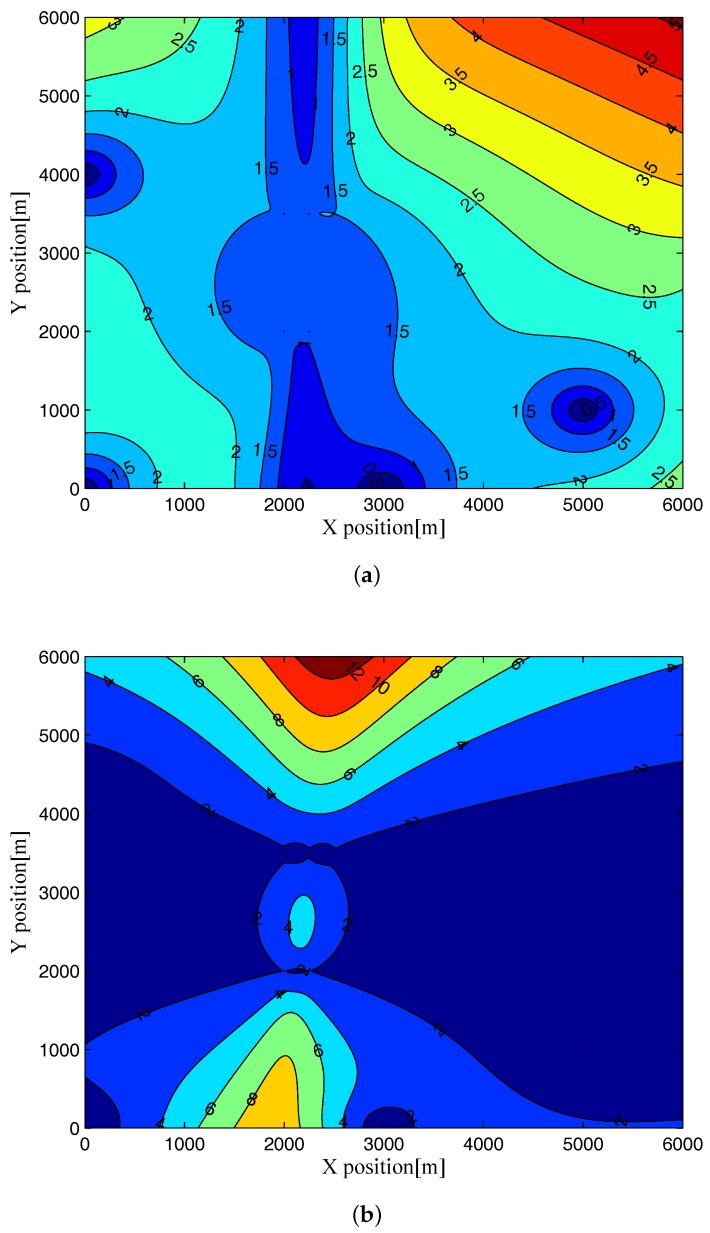
RMCRLB for target velocity dimensions in different position when SNR=0dB and $L_{r}=1000$: (**a**) x-velocity; (**b**) y-velocity.

Now, to investigate the dependence of the RMCRLB values on the geometry between the target and the passive multistatic radar system, we illustrate the RMCRLBs for both target position and velocity in different position when SNR=0dB and $L_{r}=1000$ in Figure 4 and Figure 5 . From these figures, we see that the RMCRLBs on the Cartesian coordinates of target position and velocity are different when the target is located at different positions. That is to say, as the relative geometry between the target and the multistatic radar system changes by changing the target position, the RMCRLB values are changed, which is due to the fact that the geometry between the target and the multistatic radar system impacts the derivatives of the delay-Doppler terms with respect to the Cartesian coordinates remarkably [16,34]. This will open up a new dimension for passive multistatic radar systems by aiding the optimal placement of receive stations to improve the target parameter estimation accuracy.

**Figure 6:**
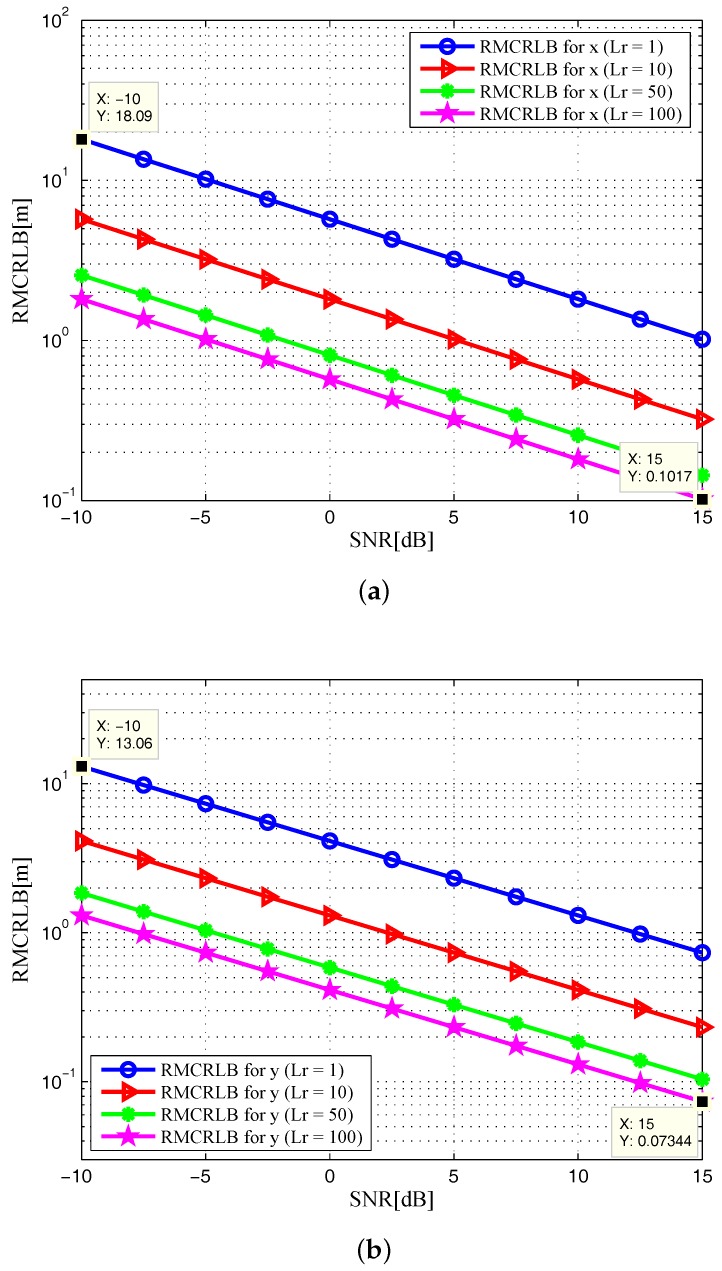
RMCRLB versus SNR in the target position dimensions with different $L_{r}$ when N=1024×512: (**a**) x-position; (**b**) y-position.

**Figure 7:**
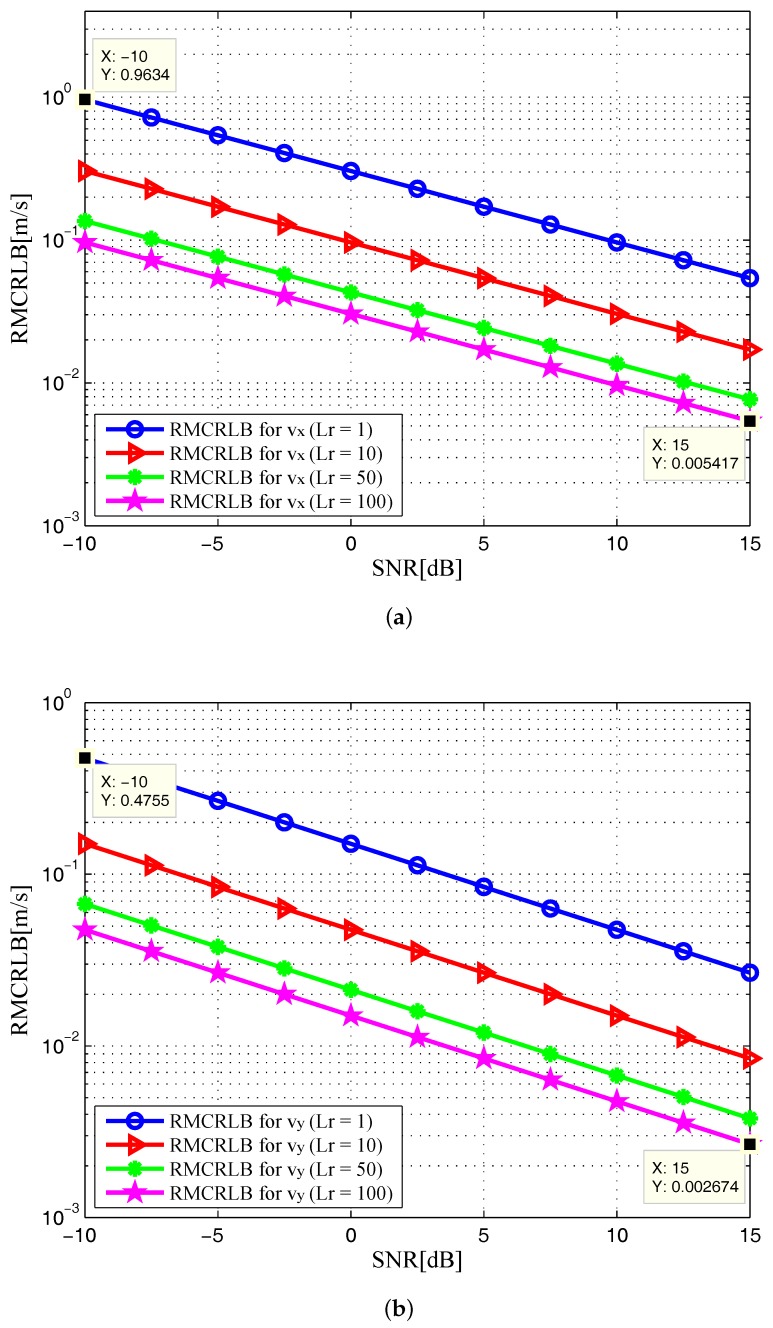
RMCRLB versus SNR in the target velocity dimensions with different $L_{r}$ when N=1024×512: (**a**) x-velocity; (**b**) y-velocity.

In Figure 6 and Figure 7, the RMCRLBs for both target position and velocity estimates are plotted as a function of SNR when N=1024×512. It can be seen that the RMCRLBs are decreased with an increase in the total number of symbols *N*. For instance, in Figure 2, at an SNR of 15dB, the RMCRLBs for the x and y positions are 0.1984m and 0.1452m when $L_{r}=100$, respectively. In Figure 3, the RMCRLBs for the x and y velocities are 2.703m/s and 1.227m/s when SNR=15dB and $L_{r}=100$, respectively. Compared the results in Figure 6, we can clearly observe that the RMCRLB at SNR=15dB for *x* is 0.1017m and that for *y* is 0.07344m. These numbers are different from the earlier case and the same holds true for the velocity RMCRLBs in Figure 7.

**Figure 8:**
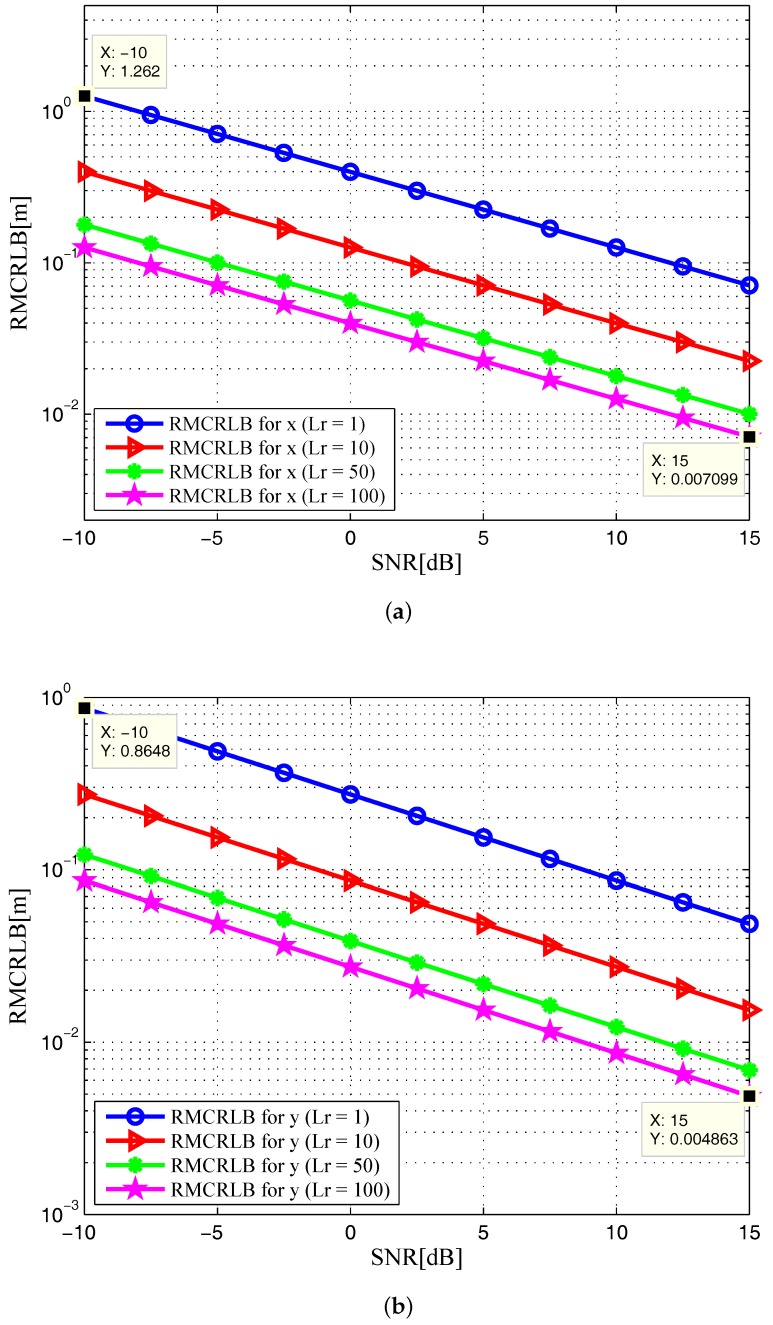
RMCRLB versus SNR in the target position dimensions with different $L_{r}$ when N=1024×512 and T=10μs: (**a**) x-position; (**b**) y-position.

**Figure 9:**
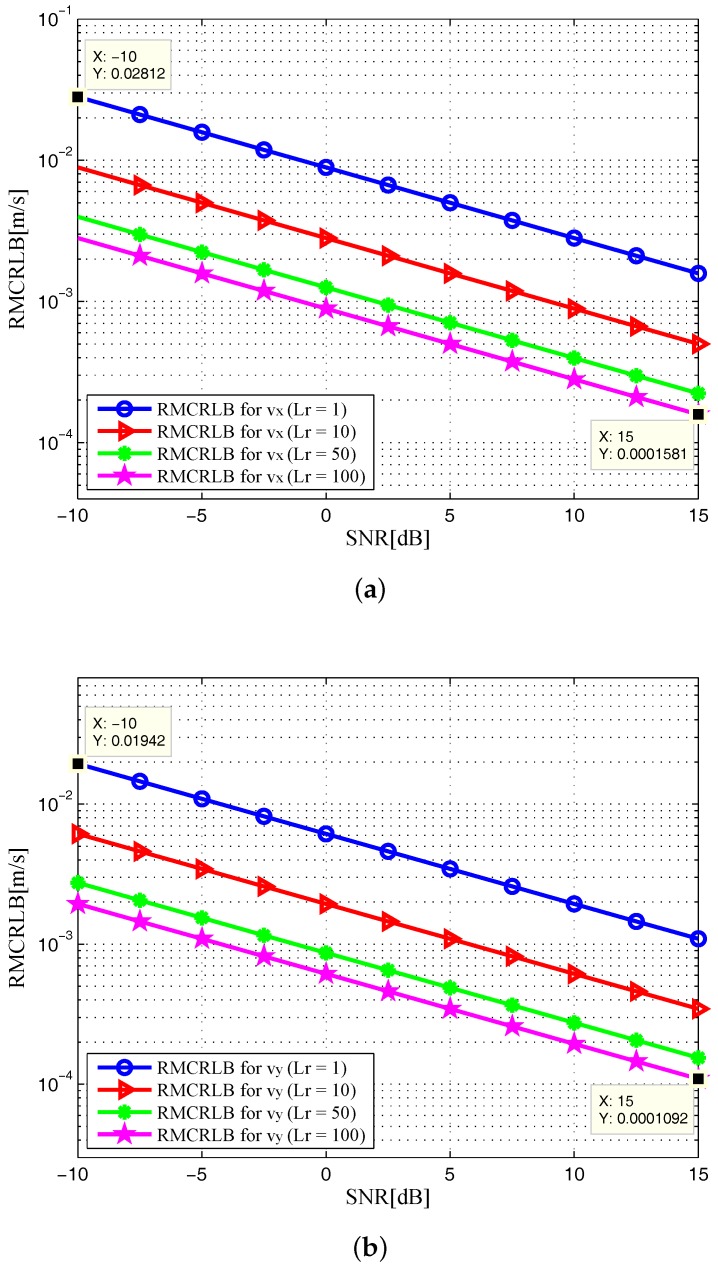
RMCRLB versus SNR in the target velocity dimensions with different $L_{r}$ when N=1024×512 and T=10μs: (**a**) x-velocity; (**b**) y-velocity.

In addition, increasing the symbol time of the signals, *T* in Equation (4) also provides benefits as one can expect. Figure 8 and Figure 9 show the RMCRLBs for both target position and velocity against SNR when N=1024×512 and T=10μs. In Figure 8, the RMCRLB value becomes 0.007099m for *x* and 0.004863m for *y* when SNR=15dB and $L_{r}=100$. Also, from Figure 9, $v_{x}$ and $v_{y}$ have much lower error values than those in Figure 7 . These results indicate that a waveform with a larger data set will achieve better estimation performance, which in turn will increase the data processing requirement. Therefore, we can conclude that the MCRLB is a function of SNR, the number of receiving antenna elements, the transmitted waveform parameters, as well as the relative geometry between the target and the passive multistatic radar configuration.

## 6. Conclusions

This paper investigates the performance of joint target position and velocity estimation employing a passive UMTS-based multistatic radar system with antenna arrays. A received signal model is developed for a multistatic radar configuration with $N_{t}$ transmit station of $L_{t}$ antenna elements and $N_{r}$ receive stations of $L_{r}$ antenna elements. The ML estimate and the MCRLB are calculated under the signal model assumptions. The derived closed-form expressions for MCRLB can be utilized to bound the target parameter estimation performance for different scenarios including a variety of radar system architectures. To provide insight, the further theoretical and numerical results are presented to demonstrate that the MCRLB not only depends on the geometry between the target and the passive multistaitc radar configuration but also depends on the SNR value and the transmitted UMTS signal parameters such as the number of symbols and symbol time. It is also shown that the joint target estimation performance of the passive multistatic radar system can be remarkably enhanced with the increase of the number of receiving elements in each receive station.

In future work, we will use this framework to study the target estimation performance of other illuminators of opportunity in addition to UMTS signals. Further, we will extend this research to the multiple-target case by appending the parameters corresponding to multiple targets into the unknown target state vector. Also, we will develop mathematical relations to obtain the optimal placement of receive stations for improving the estimation performance with arbitrary waveforms and multiple targets. 

## Figures and Tables

**Table 1:** Positions of the Transmit Stations.

Transmit Station Index	Positions [m]
TransmitStation1	[2000,2000]
TransmitStation2	[2000,2250]
TransmitStation3	[3500,2000]
TransmitStation4	[3500,2250]
TransmitStation5	[3500,2500]

**Table 2:** Positions and Moving Parameters of the Receive Stations.

Transmitter Index	Positions [m]	Velocities [m/s]
ReceiveStation1	[1000,5000]	[50,30]
ReceiveStation2	[0,0]	[80,80]
ReceiveStation3	[4000,0]	[20,90]
ReceiveStation4	[0,3000]	[100,50]
ReceiveStation5	[2000,7000]	[70,0]

## Appendix A. The Entries of Ω^1^, Ω^2^, and Ω^4^

The entries of $\Omega^{1}$, $\Omega^{2}$, and $\Omega^{4}$ are calculated as follows:

(A1) $$\begin{array}{c} \frac{\partial \tau_{ij}^{\Phi }}{\partial x}\equiv \frac{1}{c_{v}}\left(\frac{x-x_{i}^{t}}{∥\vec{p}-\vec{p_{i}^{t}}∥}+\frac{x-x_{j}^{r}}{∥\vec{p}-\vec{p_{j}^{r}}∥}\right), \end{array}$$

(A2) $$\begin{array}{c} \frac{\partial \tau_{ij}^{\Phi }}{\partial y}\equiv \frac{1}{c_{v}}\left(\frac{y-y_{i}^{t}}{∥\vec{p}-\vec{p_{i}^{t}}∥}+\frac{y-y_{j}^{r}}{∥\vec{p}-\vec{p_{j}^{r}}∥}\right), \end{array}$$

(A3) $$\begin{array}{c} \frac{\partial f_{D_{ij}}^{\Phi }}{\partial x}\equiv \frac{1}{\lambda }\left\{v_{x}\left[\frac{{\left(y-y_{i}^{t}\right)}^{2}}{{∥\vec{p}-\vec{p_{i}^{t}}∥}^{3}}+\frac{{\left(y-y_{j}^{r}\right)}^{2}}{{∥\vec{p}-\vec{p_{j}^{r}}∥}^{3}}\right]+v_{y}\left[-\frac{\left(x-x_{i}^{t}\right)\left(y-y_{j}^{r}\right)}{{∥\vec{p}-\vec{p_{i}^{t}}∥}^{3}}-\frac{\left(x-x_{j}^{r}\right)\left(y-y_{j}^{r}\right)}{{∥\vec{p}-\vec{p_{j}^{r}}∥}^{3}}\right]\right.\\ \left.+\left[v_{x,j}^{r}\frac{{\left(y-y_{j}^{r}\right)}^{2}}{∥\vec{p}-\vec{p_{j}^{r}}{∥}^{3}}-v_{y,j}^{r}\frac{\left(x-x_{j}^{r}\right)\left(y-y_{j}^{r}\right)}{∥\vec{p}-\vec{p_{j}^{r}}{∥}^{3}}\right]\right\}, \end{array}$$

(A4) $$\begin{array}{c} \frac{\partial f_{D_{ij}}^{\Phi }}{\partial y}\equiv \frac{1}{\lambda }\left\{v_{x}\left[-\frac{\left(x-x_{i}^{t}\right)\left(y-y_{j}^{r}\right)}{{∥\vec{p}-\vec{p_{i}^{t}}∥}^{3}}-\frac{\left(x-x_{j}^{r}\right)\left(y-y_{j}^{r}\right)}{{∥\vec{p}-\vec{p_{j}^{r}}∥}^{3}}\right]\right.+v_{y}\left[\frac{{\left(x-x_{i}^{t}\right)}^{2}}{{∥\vec{p}-\vec{p_{i}^{t}}∥}^{3}}+\frac{{\left(x-x_{j}^{r}\right)}^{2}}{{∥\vec{p}-\vec{p_{j}^{r}}∥}^{3}}\right]\\ \left.+\left[-v_{x,j}^{r}\frac{\left(x-x_{j}^{r}\right)\left(y-y_{j}^{r}\right)}{∥\vec{p}-\vec{p_{j}^{r}}{∥}^{3}}+v_{y,j}^{r}\frac{{\left(x-x_{j}^{r}\right)}^{2}}{∥\vec{p}-\vec{p_{j}^{r}}{∥}^{3}}\right]\right\}, \end{array}$$

(A5) $$\begin{array}{c} \frac{\partial \tau_{ij}^{\Phi }}{\partial v_{x}}\equiv 0, \end{array}$$

(A6) $$\begin{array}{c} \frac{\partial \tau_{ij}^{\Phi }}{\partial v_{y}}\equiv 0, \end{array}$$

(A7) $$\begin{array}{c} \frac{\partial f_{D_{ij}}^{\Phi }}{\partial v_{x}}\equiv \frac{1}{\lambda }\left(\frac{x-x_{i}^{t}}{∥\vec{p}-\vec{p_{i}^{t}}∥}+\frac{x-x_{j}^{r}}{∥\vec{p}-\vec{p_{j}^{r}}∥}\right), \end{array}$$

(A8) $$\begin{array}{c} \frac{\partial f_{D_{ij}}^{\Phi }}{\partial v_{y}}\equiv \frac{1}{\lambda }\left(\frac{y-y_{i}^{t}}{∥\vec{p}-\vec{p_{i}^{t}}∥}+\frac{y-y_{j}^{r}}{∥\vec{p}-\vec{p_{j}^{r}}∥}\right). \end{array}$$

## Appendix B. The Detailed Derivations of J(Ψ|c)^*UL*^ , J(Ψ|c)^*UR*^ , J(Ψ|c)^*LL*^ , and J(Ψ|c)^*LR*^

With the derivations in [17,31], we can obtain:

(A9) $$\begin{array}{cc} J{\left(\Psi |c\right)}^{UL} & =\mathrm{diag}\left\{-E_{r(t)|\Psi ,c}\left[\frac{\partial^{2}L(r(t)|\Phi ,c)}{\partial (\tau_{11}^{\Phi }{)}^{2}},\frac{\partial^{2}L(r(t)|\Phi ,c)}{\partial (\tau_{12}^{\Phi }{)}^{2}},\cdots ,\frac{\partial^{2}L(r(t)|\Phi ,c)}{\partial (\tau_{N_{t}N_{r}}^{\Phi }{)}^{2}}\right]\right\}\\ & =8\pi^{2}\mathrm{diag}\left(\frac{L_{r}EG^{2}{\left|\xi_{11}\right|}^{2}}{N_{t}L_{t}\sigma_{w}^{2}},\frac{L_{r}EG^{2}{\left|\xi_{12}\right|}^{2}}{N_{t}L_{t}\sigma_{w}^{2}},\cdots ,\frac{L_{r}EG^{2}{\left|\xi_{N_{t}N_{r}}\right|}^{2}}{N_{t}L_{t}\sigma_{w}^{2}}\right)⊙\left\{I_{N_{r}}⊗\mathrm{diag}\left(\epsilon_{1},\epsilon_{2},\cdots ,\epsilon_{N_{t}}\right)\right\}, \end{array}$$

where $I_{N_{r}}$ denotes an $N_{r}\times N_{r}$ identity matrix. The term $\epsilon_{i}$ is given by:

(A10) $$\begin{array}{cc} \epsilon_{i} & \equiv E\left\{\int_{-\infty }^{+\infty }f^{2}{\left|U_{i}\left(f\right)\right|}^{2}df-{\left|\int_{-\infty }^{+\infty }f{\left|U_{i}\left(f\right)\right|}^{2}df\right|}^{2}\right\}=\frac{1}{12T^{2}}\left(1+3\alpha^{2}-24\frac{\alpha^{2}}{\pi^{2}}\right). \end{array}$$

Similarly, we have

(A11) $$\begin{array}{cc} J{\left(\Psi |c\right)}^{UR}=J{\left(\Psi |c\right)}^{LL} & =\mathrm{diag}\left\{-E_{r(t)|\Psi ,c}\left[\frac{\partial^{2}L(r(t)|\Phi ,c)}{\partial \tau_{11}^{\Phi }f_{D_{11}}^{\Phi }},\frac{\partial^{2}L(r(t)|\Phi ,c)}{\partial \tau_{12}^{\Phi }f_{D_{12}}^{\Phi }},\cdots ,\frac{\partial^{2}L(r(t)|\Phi ,c)}{\partial \tau_{N_{t}N_{r}}^{\Phi }f_{D_{N_{t}N_{r}}}^{\Phi }}\right]\right\}\\ & =8\pi^{2}\mathrm{diag}\left(\frac{L_{r}EG^{2}{\left|\xi_{11}\right|}^{2}}{N_{t}L_{t}\sigma_{w}^{2}},\frac{L_{r}EG^{2}{\left|\xi_{12}\right|}^{2}}{N_{t}L_{t}\sigma_{w}^{2}},\cdots ,\frac{L_{r}EG^{2}{\left|\xi_{N_{t}N_{r}}\right|}^{2}}{N_{t}L_{t}\sigma_{w}^{2}}\right)⊙\mathrm{diag}\left(\gamma_{11},\gamma_{12},\cdots ,\gamma_{N_{t}N_{r}}\right), \end{array}$$

where

(A12) $$\gamma_{ij}\equiv E\left\{\frac{1}{2\pi }ℑ\left[\int_{-\infty }^{+\infty }tu_{i}^{*}\left(t-\tau_{ij}^{\Phi }\right)\frac{\partial u_{i}\left(t-\tau_{ij}^{\Phi }\right)}{\partial \tau_{ij}^{\Phi }}dt\right]-\int_{-\infty }^{+\infty }f{\left|U_{i}\left(f\right)\right|}^{2}df\int_{-\infty }^{+\infty }t{\left|u_{i}\left(t-\tau_{ij}^{\Phi }\right)\right|}^{2}dt\right\}=0,$$

and

(A13) $$\begin{array}{cc} J{\left(\Psi |c\right)}^{LR} & =\mathrm{diag}\left\{-E_{r(t)|\Psi ,c}\left[\frac{\partial^{2}L(r(t)|\Phi ,c)}{\partial {\left(f_{D_{11}}^{\Phi }\right)}^{2}},\frac{\partial^{2}L(r(t)|\Phi ,c)}{\partial {\left(f_{D_{12}}^{\Phi }\right)}^{2}},\cdots ,\frac{\partial^{2}L(r(t)|\Phi ,c)}{\partial {\left(f_{D_{N_{t}N_{r}}}^{\Phi }\right)}^{2}}\right]\right\}\\ & =8\pi^{2}\mathrm{diag}\left(\frac{L_{r}EG^{2}{\left|\xi_{11}\right|}^{2}}{N_{t}L_{t}\sigma_{w}^{2}},\frac{L_{r}EG^{2}{\left|\xi_{12}\right|}^{2}}{N_{t}L_{t}\sigma_{w}^{2}},\cdots ,\frac{L_{r}EG^{2}{\left|\xi_{N_{t}N_{r}}\right|}^{2}}{N_{t}L_{t}\sigma_{w}^{2}}\right)⊙\mathrm{diag}\left(\eta_{11},\eta_{12},\cdots ,\eta_{N_{t}N_{r}}\right), \end{array}$$

where

(A14) $$\eta_{ij}\equiv E\left\{\int_{-\infty }^{+\infty }t^{2}{\left|u_{i}\left(t-\tau_{ij}^{\Phi }\right)\right|}^{2}df-{\left|\int_{-\infty }^{+\infty }t{\left|u_{i}\left(t-\tau_{ij}^{\Phi }\right)\right|}^{2}dt\right|}^{2}\right\}=\frac{T^{2}}{4}\left(\frac{1}{4\alpha }+\frac{N^{2}-1}{3}\right).$$

From Equations (A10), (A12), and (A14), we can notice that the terms $\epsilon_{i}$, $\gamma_{ij}$, and $\eta_{ij}$ are dependent on the characteristics of the UMTS waveforms.

## Appendix C. The Elements of FIM J_*ij*_ (Φ|c)

The elements of the symmetric FIM $J_{ij}\left(\Phi |c\right)$ corresponding to the ij-th transmit-to-receive path are given by:

(A15) $$\begin{array}{cc} J_{ij}^{11}\left(\Phi |c\right) & =\frac{1}{12T^{2}}\left(1+3\alpha^{2}-24\frac{\alpha^{2}}{\pi^{2}}\right){\left(\frac{\partial \tau_{ij}^{\Phi }}{\partial x}\right)}^{2}+\frac{T^{2}}{4}\left(\frac{1}{4\alpha }+\frac{N^{2}-1}{3}\right){\left(\frac{\partial f_{D_{ij}}^{\Phi }}{\partial x}\right)}^{2}, \end{array}$$

(A16) $$\begin{array}{cc} J_{ij}^{12}\left(\Phi |c\right)=J_{ij}^{21}\left(\Phi |c\right) & =\frac{1}{12T^{2}}\left(1+3\alpha^{2}-24\frac{\alpha^{2}}{\pi^{2}}\right)\left(\frac{\partial \tau_{ij}^{\Phi }}{\partial x}\right)\left(\frac{\partial \tau_{ij}^{\Phi }}{\partial y}\right)\\ & +\frac{T^{2}}{4}\left(\frac{1}{4\alpha }+\frac{N^{2}-1}{3}\right)\left(\frac{\partial f_{D_{ij}}^{\Phi }}{\partial x}\right)\left(\frac{\partial f_{D_{ij}}^{\Phi }}{\partial y}\right), \end{array}$$

(A17) $$\begin{array}{cc} J_{ij}^{13}\left(\Phi |c\right) & =J_{ij}^{31}\left(\Phi |c\right)=\frac{T^{2}}{4}\left(\frac{1}{4\alpha }+\frac{N^{2}-1}{3}\right)\left(\frac{\partial f_{D_{ij}}^{\Phi }}{\partial x}\right)\left(\frac{\partial f_{D_{ij}}^{\Phi }}{\partial v_{x}}\right), \end{array}$$

(A18) $$\begin{array}{cc} J_{ij}^{14}\left(\Phi |c\right) & =J_{ij}^{41}\left(\Phi |c\right)=\frac{T^{2}}{4}\left(\frac{1}{4\alpha }+\frac{N^{2}-1}{3}\right)\left(\frac{\partial f_{D_{ij}}^{\Phi }}{\partial x}\right)\left(\frac{\partial f_{D_{ij}}^{\Phi }}{\partial v_{y}}\right), \end{array}$$

(A19) $$\begin{array}{cc} J_{ij}^{22}\left(\Phi |c\right) & =\frac{1}{12T^{2}}\left(1+3\alpha^{2}-24\frac{\alpha^{2}}{\pi^{2}}\right){\left(\frac{\partial \tau_{ij}^{\Phi }}{\partial y}\right)}^{2}+\frac{T^{2}}{4}\left(\frac{1}{4\alpha }+\frac{N^{2}-1}{3}\right){\left(\frac{\partial f_{D_{ij}}^{\Phi }}{\partial y}\right)}^{2}, \end{array}$$

(A20) $$\begin{array}{cc} J_{ij}^{23}\left(\Phi |c\right) & =J_{ij}^{32}\left(\Phi |c\right)=\frac{1}{12T^{2}}\left(1+3\alpha^{2}-24\frac{\alpha^{2}}{\pi^{2}}\right)\left(\frac{\partial f_{D_{ij}}^{\Phi }}{\partial y}\right)\left(\frac{\partial f_{D_{ij}}^{\Phi }}{\partial v_{x}}\right), \end{array}$$

(A21) $$\begin{array}{cc} J_{ij}^{24}\left(\Phi |c\right) & =J_{ij}^{42}\left(\Phi |c\right)=\frac{1}{12T^{2}}\left(1+3\alpha^{2}-24\frac{\alpha^{2}}{\pi^{2}}\right)\left(\frac{\partial f_{D_{ij}}^{\Phi }}{\partial y}\right)\left(\frac{\partial f_{D_{ij}}^{\Phi }}{\partial v_{y}}\right), \end{array}$$

(A22) $$\begin{array}{cc} J_{ij}^{33}\left(\Phi |c\right) & =\frac{T^{2}}{4}\left(\frac{1}{4\alpha }+\frac{N^{2}-1}{3}\right){\left(\frac{\partial f_{D_{ij}}^{\Phi }}{\partial v_{x}}\right)}^{2}, \end{array}$$

(A23) $$\begin{array}{cc} J_{ij}^{34}\left(\Phi |c\right)=J_{ij}^{43}\left(\Phi |c\right) & =\frac{T^{2}}{4}\left(\frac{1}{4\alpha }+\frac{N^{2}-1}{3}\right)\left(\frac{\partial f_{D_{ij}}^{\Phi }}{\partial v_{x}}\right)\left(\frac{\partial f_{D_{ij}}^{\Phi }}{\partial v_{y}}\right), \end{array}$$

(A24) $$\begin{array}{cc} J_{ij}^{44}\left(\Phi |c\right) & =\frac{T^{2}}{4}\left(\frac{1}{4\alpha }+\frac{N^{2}-1}{3}\right){\left(\frac{\partial f_{D_{ij}}^{\Phi }}{\partial v_{y}}\right)}^{2}. \end{array}$$

## References

[1] Li J., Stoica P. (2009). MIMO Radar Signal Processing.

[2] Fisher E., Haimovich A., Blum R.S., Cimini L.J., Chizhik D., Valenzuela R. (2006). Spatial diversity in radars—Models and detection performance. IEEE Trans. Signal Process..

[3] Haimovich A.M., Blum R.S., Cimini L.J. (2008). MIMO radar with widely separated antennas. IEEE Signal Process. Mag..

[4] Chen P., Zheng L., Wang X.D., Li H.B., Wu L.N. (2017). Moving target detection using colocated MIMO radar on multiple distributed moving platforms. IEEE Trans. Signal Process..

[5] Naghsh M.M., Mahmoud M.H., Shahram S.P., Soltanalian M., Stoica P. (2013). Unified optimization framework for multi-static radar code design using information-theoretic criteria. IEEE Trans. Signal Process..

[6] Niu R.X., Blum R.S., Varshney P.K., Drozd A.L. (2010). Target localization and tracking in noncoherent multiple-input multiple-output radar systems. IEEE Trans. Aerosp. Electron. Syst..

[7] Godrich H., Petropulu A.P., Poor H.V. (2011). Power allocation strategies for target localization in distributed multiple-radar architectures. IEEE Trans. Signal Process..

[8] Khomchuk P., Blum R.S., Bilik I. (2016). Performance analysis of target parameters estimation using multiple widely separated antenna arrays. IEEE Trans. Aerosp. Electron. Syst..

[9] Godrich H., Haimovich A.M., Blum R.S. (2010). Target localization accuracy gain in MIMO radar-based systems. IEEE Trans. Inf. Theory.

[10] He Q., Blum R.S., Haimovich A.M. (2010). Noncoherent MIMO radar for location and velocity estimation: More antennas means better performance. IEEE Trans. Signal Process..

[11] He Q., Blum R.S., Godrich H., Haimovich A.M. (2010). Target velocity estimation and antenna placement for MIMO radar with widely separated antennas. IEEE J. Sel. Top. Signal Process..

[12] He Q., Blum R.S. (2012). Noncoherent versus coherent MIMO radar: Performance and simplicity analysis. Signal Process..

[13] Wei C., He Q., Blum R.S. Cramer-Rao bound for joint location and velocity estimation in multi-target non-coherent MIMO radars. Proceedings of the 2010 44th Annual Conference on Information Sciences and Systems (CISS).

[14] He Q., Blum R.S., Haimovich A.M. (2016). Generalized Cramér-Rao bound for joint estimation of target position and velocity for active and passive radar networks. IEEE Trans. Signal Process..

[15] Zhao T., Huang T.Y. (2016). Cramer-Rao lower bounds for the joint delay-Doppler estimation of an extended extended target. IEEE Trans. Signal Process..

[16] Shi C.G., Salous S., Wang F., Zhou J.J. (2016). Cramer-Rao lower bound evaluation for linear frequency modulation based active radar networks operating in a Rice fading environment. Sensors.

[17] Cheng Z.Y., He Z.S., Zheng X.J. CRB for joint estimation of moving target in distributed phased array radars on moving platforms. Proceedings of the IEEE 13th International Conference on Signal Processing (ICSP).

[18] Ferreol A., Larzabal M., Viberg M. (2006). On the asymptotic performance analysis of subspace DOA estimation in the presence of modeling errors: Case of MUSIC. IEEE Trans. Signal Process..

[19] Ciuonzo D., Romano G., Solimene R. (2015). Performance analysis of time-reversal MUSIC. IEEE Trans. Signal Process..

[20] Ciuonzo D., Romano G., Solimene R. On MSE performance of time-reversal MUSIC. Proceedings of the 2014 IEEE 8th Sensor Array and Multichannel Signal Processing Workshop (SAM).

[21] Hack D.E., Patton L.K., Himed B., Saville M.A. (2014). Detection in passive MIMO radar networks. IEEE Trans. Signal Process..

[22] Zaimbashi A. (2017). Target detection in analog terrestrial TV-based passive radar sensor: Joint delay-Doppler estimation. IEEE Sens. J..

[23] Greco M.S., Stinco P., Gini F., Farina A. (2011). Cramer-Rao bounds and selection of bistatic channels for multistatic radar systems. IEEE Trans. Aerosp. Electron. Syst..

[24] Pace P.E. (2009). Detecting and Classifying Low Probability of Intercept Radar.

[25] Shi C.G., Salous S., Wang F., Zhou J.J. (2017). Power allocation for target detection in radar networks based on low probability of intercept: A cooperative game theoretical strategy. Radio Sci..

[26] Zhang Z.K., Tian Y.B. (2016). A novel resource scheduling method of netted radars based on Markov decision process during target tracking in clutter. EURASIP J. Adv. Signal Process..

[27] Shi C.G., Zhou J.J., Wang F. LPI based resource management for target tracking in distributed radar network. Proceedings of the 2015 IEEE International Conference on Signal Processing, Communications and Computing (ICSPCC).

[28] Shi C.G., Wang F., Sellathurai M., Zhou J.J. (2017). Low probability of intercept based multicarrier radar jamming power allocation for joint radar and wireless communications systems. IET Radar Sonar Navig..

[29] Li N.J. (1995). Radar ECCMS new area: Anti-stealth and anti-ARM. IEEE Trans. Aerosp. Electron. Syst..

[30] Shi C.G., Wang F., Zhou J.J. (2016). Cramér-Rao bound analysis for joint target position and velocity estimation in FM-based passive radar networks. IET Signal Process..

[31] Gogineni S., Rangaswamy M., Rigling B.D., Nehorai A. (2014). Cramér-Rao bounds for UMTS-based passive multistatic radar. IEEE Trans. Signal Process..

[32] Javed M.N., Ali S., Hassan S.A. (2016). 3D MCRLB evaluation of a UMTS-based passive multistatic radar operating in a line-of-sight environment. IEEE Trans. Signal Process..

[33] Filip A., Shutin D. (2016). Cramer-Rao bounds for L-band digital aeronautical communication system type 1 based passive multiple-input multiple-output radar. IET Radar Sonar Navig..

[34] Shi C.G., Salous S., Wang F., Zhou J.J. (2017). Modified Cramér-Rao lower bounds for joint position and velocity estimation of a Rician target in OFDM-based passive radar networks. Radio Sci..

[35] Shi C.G., Wang F., Sellathurai M., Zhou J.J. (2016). Transmitter subset selection in FM-based passive radar networks for joint target parameter estimation. IEEE Sensors J..

[36] Xie R., Wan X.R., Hong S., Yi J.X. (2017). Joint optimization of receiver placement and illuminator selection for a multiband passive radar network. Sensors.

[37] He Q., Blum R.S. (2014). The significant gains from optimally processed multiple signals of opportunity and multiple receive stations in passive radar. IEEE Signal Process. Lett..

[38] Jardak S., Ahmed S., Alouini M.S. Low complexity joint estimation of reflection coefficient, spatial location, and Doppler shift for MIMO-radar by exploiting 2D-FFT. Proceedings of the 2014 International Radar Conference (Radar).

[39] Tian J., Cui W., Lv X.L., Wu S., Hou J.G., Wu S.L. (2014). Joint estimation algorithm for multi-targets’ motion parameters. IET Radar Sonar Navig..

